# Hallmarks of the pre-disease state: prevention and control of the pre-disease state, a tipping point between health and disease

**DOI:** 10.1038/s41421-026-00879-4

**Published:** 2026-03-03

**Authors:** Pei Chen, Xiaoping Liu, Rui Liu, Luonan Chen

**Affiliations:** 1 School of Mathematics, South China University of Technology, Guangzhou, Guangdong China; 2 Key Laboratory of Systems Health Science of Zhejiang Province, School of Life Science, Hangzhou Institute for Advanced Study, University of Chinese Academy of Sciences, Hangzhou, Zhejiang China; 3 School of Mathematical Sciences and School of AI, Shanghai Jiao Tong University, Shanghai, China

Complex diseases, including cancers, diabetes, and other chronic diseases, are not caused by individual genes or factors. Instead, they arise from dynamic interactions among multiple genetic, environmental, and lifestyle factors. Disease pathogenesis is a prolonged, dynamically evolving process, not a simple binary switch between health and disease. Between the states of health and manifest disease lies a crucial yet often overlooked intermediate phase: the pre-disease state, i.e., a tipping point that occurs just before the disease state^1^. This phase determines whether a disease will develop, when it might occur, and importantly, whether the disease process can be reversed. Preventive intervention during this stage, which occurs prior to disease onset, is therefore crucial for effective disease management and potential cure.

The concept of “*Wei-Bing*,” also referred to as the pre-disease state, originates from traditional Chinese medicine (TCM) and represents a specific health state or heightened susceptibility prior to the onset of disease. As the tipping point between normal and disease states, it is thought to be the best period for preventive treatment of complex diseases. Treatment of *Wei-Bing*, or preventive treatment of disease, was proposed in ancient China approximately 2000 years ago and aims to intervene in the *Wei-Bing* state in order to treat diseases before their onset^2^. As shown in Fig. 1a, the continuum of disease progression can be understood as a dynamic process with a three-state framework: “normal state → pre-disease state → disease state”^3^. Notably, the pre-disease state/*Wei-Bing* is an extreme of the normal state and shares many of its characteristics, making the differentiation between these two states subtle and complex. Historically, TCM has approached disease progression primarily through individual phenotypic observations and empirical knowledge (Fig. 1a). However, a lack of quantitative measures for pre-disease states has limited the integration of this concept into statistics-based or evidence-based medicine, thereby hindering the development of preventive treatment strategies.

**Fig. 1:**
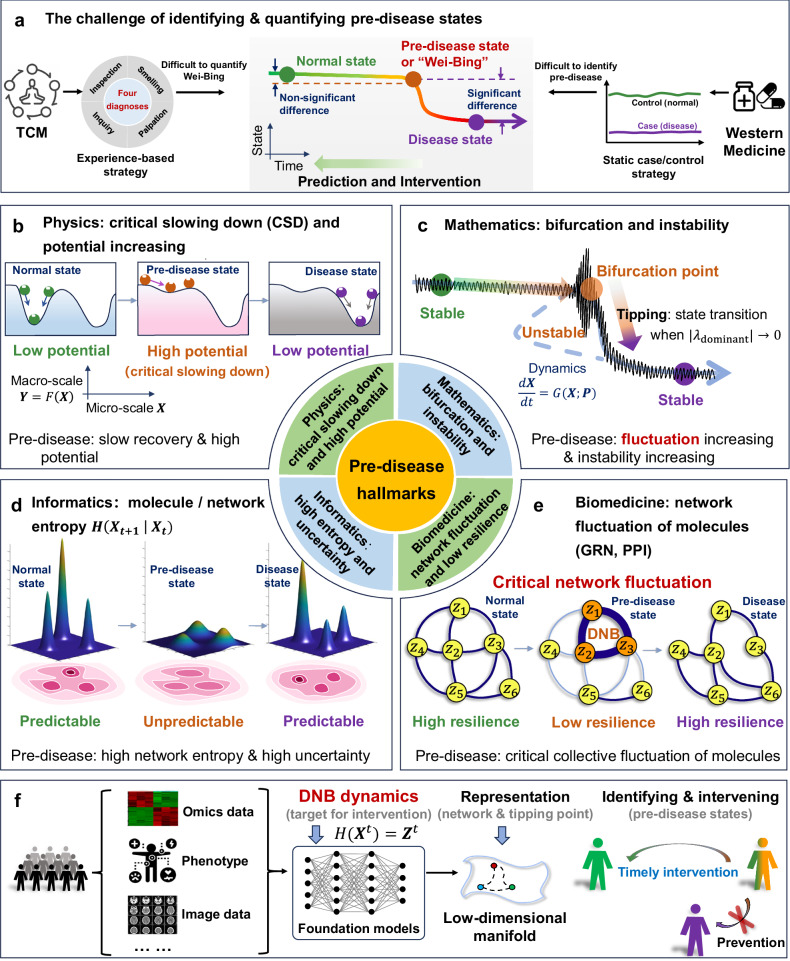
Hallmarks and interdisciplinary framework of the pre-disease (*Wei-Bing*) state. **a** Conceptual challenge in identification and quantification of the pre-disease state. TCM lacks quantitative criteria, whereas static case–control paradigms in Western medicine have difficulty identifying pre-disease samples that exhibit non-significant differences from healthy controls. **b**–**e** Hallmarks of the pre-disease state across disciplines: Physics: critical slowing down (CSD) and increasing potential (**b**); Mathematics: proximity to bifurcation and dynamical instability (**c**); Informatics: increased molecular or network entropy and uncertainty (**d**); Biomedicine: collective network fluctuation and reduced resilience (**e**). **f** AI-based foundation models with dynamics-based approaches can identify the pre-disease state by integrating multi-modal/multi-scale data, thus revealing DNBs and supporting timely interventions.

In contemporary Western medicine, molecular and network biomarkers^4^ can effectively distinguish disease samples (disease state) from those of healthy individuals (normal state), leveraging a static case/control strategy. However, these biomarkers are less effective at identifying pre-disease samples, in which differences from the healthy state are typically not significant^3^ (Fig. 1a). This often results in misclassification of pre-disease states as normal states, making it difficult to identify the *Wei-Bing* stage.

This limitation highlights the need for new theoretical frameworks that define and quantify the pre-disease state from a dynamic rather than a static perspective. We propose that the pre-disease state can be systematically characterized by a set of hallmarks that reflect its underlying criticality. These hallmarks emerge consistently across multiple disciplines and together define an unstable but potentially reversible system state that precedes disease onset.

## Hallmark 1 (physics): critical slowing down (CSD) and increasing potential

From a physics perspective, the disease progression of an organ or individual can be viewed as motion in a potential landscape^5,6^. The healthy state corresponds to a low potential, with rapid recovery from perturbations. By contrast, the pre-disease state is associated with a high-potential landscape and CSD, whereby recovery from perturbations becomes progressively slower despite the absence of overt disease.

Notably, the signatures of the pre-disease state exhibit scale dependence. At the microscale, as represented by state variables $x$ (e.g., gene/protein expression profiles), early hallmarks manifest as enhanced local fluctuations in these molecular components and increased sensitivity to external or internal perturbations. By contrast, at the macroscale, corresponding to derived phenotypic traits ${\boldsymbol{Y}}$ (e.g., clinical indicators or systemic physiological states), i.e., ${\boldsymbol{Y}}=F({\boldsymbol{X}})$ where$\,F({\boldsymbol{X}})$ denotes the mapping from microscale molecular states to macroscale phenotypes, the system dynamics of ${\boldsymbol{Y}}$ gradually become decoupled from ${\boldsymbol{X}}$. As a result, ${\boldsymbol{Y}}$ exhibits reduced fluctuation amplitude compared with ${\boldsymbol{X}}$ and diminished sensitivity to perturbations of ${\boldsymbol{X}}$. Such scale-dependent divergence underscores the challenge of detecting pre-disease states using only macroscale phenotypic data, as early microscale dysregulations are often “averaged out” at the systemic level.

## Hallmark 2 (mathematics): proximity to bifurcation and dynamical instability

From a mathematical perspective, the pre-disease state corresponds to a system approaching a bifurcation, in which stability progressively decreases^5^. Disease progression, described by $\frac{d{\boldsymbol{X}}}{{dt}}={G}({\boldsymbol{X}};{\boldsymbol{P}})$, can be viewed as the gradual evolution of system parameters ${\boldsymbol{P}}$ (e.g., environmental factors, genetic mutations, and lifestyle factors) toward a bifurcation point, under which a previously stable attractor loses stability as the dominant eigenvalue of the Jacobian matrix of $G$ approaches zero. In such a state ${\boldsymbol{X}}$, the influence of small perturbations increases, rendering the system highly sensitive to perturbations, i.e., exhibiting strong fluctuations in ${\boldsymbol{X}}$.

Therefore, the pre-disease state is a specific stable phase that occurs just before a bifurcation/critical point or before the loss of stability. Once this point is crossed, the system undergoes a qualitative state transition and settles into an alternative stable state that corresponds to the disease state.

## Hallmark 3 (informatics): increased molecule/network entropy and uncertainty

From an informatics perspective, the pre-disease state is characterized by increased molecule/network entropy and uncertainty in system dynamics ${\boldsymbol{X}}$. Rather than predictable and well-constrained attractors as in the normal or disease states, the pre-disease state exhibits weakened temporal dependencies and increased variability in state evolution, leading to a significant loss in predictability (information/entropy $H$) of future system behavior.

Importantly, such an increase in entropy does not simply reflect random noise, but arises from underlying dynamical instability near critical transitions. Consequently, early-warning signals in the pre-disease state are encoded in distributional changes rather than static state differences, which can be quantified using metrics such as network flow entropy $H({{\boldsymbol{X}}}_{t+1}{|}{{\boldsymbol{X}}}_{t})$^7^ or optimal transport distances^8^.

## Hallmark 4 (biomedicine): collective network fluctuation and reduced resilience

From a biomedical perspective, the pre-disease state is characterized by critical collective fluctuations within molecular interaction networks such as gene regulatory networks (GRNs) and protein–protein interaction (PPI) networks. Rather than isolated dysregulation of individual molecules, a subset of network components exhibits high fluctuations and strong correlations in dynamics, forming dynamic network biomarkers (DNBs)^3^. These collective network-level fluctuations are a hallmark of biological systems approaching a disease deterioration/transition.

In contrast to the relatively stable networks of healthy and disease states, the pre-disease state exhibits decreased network resilience, whereby collective molecular fluctuations signal an imminent shift toward a disease state. This network-centric view supports the use of DNBs in biomedical applications^9,10^.

These hallmarks imply that, in contrast to identifying a disease state by traditional “differential studies” of molecular concentrations, quantification of a pre-disease state or prediction of a disease state requires new “fluctuation studies” of molecular concentrations. Unlike current static “case/control studies” in biology or medicine, quantification of tipping points provides a new approach for dynamical “process studies” of complex biological processes. Effective early-warning methods for *Wei-Bing* states have been developed from time-series or cross-sectional omics data^3,7,8^. In addition, AI methods have been used to predict the risk of complex diseases and to identify diseases by integrating multi-scale and multi-modal data^11^; in this case, the original high-dimensional dynamics ${X}^{t}={({x}_{1}^{t},{x}_{2}^{t},\ldots ,{x}_{n}^{t})}^{{\rm{T}}}$ may be represented with a latent low-dimensional state ${Y}^{t}={({y}_{1}^{t},{y}_{2}^{t},\ldots ,{y}_{m}^{t})}^{{\rm{T}}}$, i.e., $\Phi ({X}^{t})={Y}^{t}$, which is a static representation at time point $t$. Conventional deep learning models treat each time step as an independent sample and focus on capturing global dependencies, making them well suited for long-sequence analysis. Thus, these static states are well suited to representing statistical levels of clinical/biological markers for disease diagnosis, rather than pre-disease identification.

According to the CSD theory, when a dynamical system approaches a bifurcation point from a stable steady state, the space is generically constrained to a center manifold, which typically has codim-1 local bifurcation with a dominant real eigenvalue^5^. In addition, on the basis of the generalized Takens’ embedding theorem^12^, the dynamics of a system can be topologically reconstructed from the delay embedding scheme under certain conditions, i.e., the spatiotemporal information (STI) transformation converts the original spatial information of high-dimensional data ${X}^{t}={({x}_{1}^{t},{x}_{2}^{t},\ldots ,{x}_{n}^{t})}^{{\rm{T}}}$ into the temporal dynamics ${Z}^{t}=\left({z}^{t},{z}^{t+1},\cdots ,{z}^{t+L-1}\right)$ of a one-dimensional delay embedding of this single variable $y$ by $H({X}^{t})={Z}^{t}$, where $L$ represents the embedding dimension. Different from the static representation ${Y}^{t}$, this dynamical representation ${Z}^{t}$ from $t$ to $t+1,\ldots ,t+L-1$ can be roughly considered to represent the (low-dimensional) center manifold near the tipping point and exploits both inter-sample (cross-sample) information and intra-sample information embedded in high-dimensional/spatial data^13^.

By integrating AI-based methods with dynamics-based methods, such as the STI approach, a foundation or agentic model can be trained using large-scale multimodal data to precisely quantify the state of organs or tissues during disease progression (Fig. 1f). Such a foundation model can be pretrained on large-scale public clinical databases such as MIMIC, eICU, and HiRID, as well as institution-specific medical cohorts, to capture universal dynamical patterns of pre-disease states. Once pretrained, the model can be adapted to new diseases or data types by fine-tuning on task-specific datasets or applied directly in a zero-shot setting when labeled data are limited. This paradigm enables the transfer of dynamical knowledge across diseases, organs, and populations, thereby providing a scalable and data-efficient framework for identification of pre-disease states.

In addition to detection of pre-disease states, another important but less explored question is whether disease progression can be altered by active intervention before critical transitions. Theoretically, disease progression should be reversible by intervention at the tipping point before transition to the disease state. Conceptually, intervention at this stage does not aim to reverse established disease, but rather to causally perturb key regulatory mechanisms to prevent the system from crossing an irreversible tipping point, thereby restoring the system to the normal state. For example, in a Kras–Lkb1 mutant lung cancer model, temporal genetic and pharmacological perturbations of the Wnt pathway showed that adeno-to-squamous transdifferentiation could be suppressed when intervention was applied before, but not after, the critical transition^14^. This observation highlights the importance of intervention timing in relation to the underlying causal dynamics of the system, rather than merely its observable phenotypic state. Identifying causal factors and intervening before critical transitions have the potential to reduce disease burden, alleviate long-term healthcare demands, and lower the overall cost of disease management, thereby driving a paradigm shift in medicine from passive treatment to active prevention.

Identifying the pre-disease or *Wei-Bing* state on the basis of specific hallmarks is a prerequisite and fundamental step for prevention of complex diseases, but several key challenges remain to be addressed. First, for some acute diseases such as acute myocardial infarction, stroke, and acute organ failure, pre-disease or out-of-hospital data are inherently difficult to obtain in conventional clinical settings because early pathological changes are often clinically silent, delaying clinical presentation and routine data collection. In this context, the development of continuous sensing technologies may provide a practical avenue for capturing the early physiological signals that precede critical transitions. Second, although existing applications illustrate the importance of intervention timing, we still lack systematic algorithms/models explicitly designed to capture causal dynamics and predict intervention outcomes near critical transitions; such models warrant further investigation. Third, addressing these challenges will require the development of effective dynamical models, particularly AI-based models capable of integrating multi-modal data, including phenotypic information, imaging data, and omics data arising from biological and environmental factors. Lastly, the hallmarks discussed here are not intended to be exhaustive; additional hallmarks of the pre-disease state may emerge as future studies further refine and validate this framework.

Owing to the importance of such interdisciplinary pre-disease studies, the National Natural Science Foundation of China has initiated a series of special projects since 2022 focused on quantifying the *Wei-Bing* state across multiple disciplines and hallmarks (https://www.ncsti.gov.cn/kjdt/tzgg/202211/t20221103_101836.html). Similarly, the major project “Ultra early disease prediction and intervention” was established under the Scientific Moonshot R&D program (https://www.jst.go.jp/moonshot/en/) of the Japan Science and Technology Agency in 2019. The National Institutes of Health in the USA also initiated a project for assessment and management of disease risk in 2020 (https://www.genome.gov/news/news-release/NIH-funds-centers-to-improve-role-of-genomics-in-assessing-and-managing-disease-risk), and the Medical Research Council of the UK launched a project supporting early-stage development of new healthcare interventions (https://www.ukri.org/opportunity/funding-for-early-stage-development-of-new-healthcare-interventions/). These national projects are expected to further promote interdisciplinary studies aimed at accurate quantification of pre-disease states/*Wei-Bing*, paving the way for preventive treatments in both TCM and Western medicine.
